# Measuring metacognitive performance: type 1 performance dependence and test-retest reliability

**DOI:** 10.1093/nc/niab040

**Published:** 2021-11-25

**Authors:** Matthias Guggenmos

**Affiliations:** Department of Psychiatry and Neurosciences, Charité – Universitätsmedizin Berlin, corporate member of Freie Universität Berlin and Humboldt-Universität zu Berlin, Charitéplatz 1, Berlin 10117, Germany

**Keywords:** metacognitionl, confidencel, decision makingl, metacognitive sensitivitl, test-retest reliability

## Abstract

Research on metacognition—thinking about thinking—has grown rapidly and fostered our understanding of human cognition in healthy individuals and clinical populations. Of central importance is the concept of metacognitive performance, which characterizes the capacity of an individual to estimate and report the accuracy of primary (type 1) cognitive processes or actions ensuing from these processes. Arguably one of the biggest challenges for measures of metacognitive performance is their dependency on objective type 1 performance, although more recent methods aim to address this issue. The present work scrutinizes the most popular metacognitive performance measures in terms of two critical characteristics: independence of type 1 performance and test-retest reliability. Analyses of data from the Confidence Database (total *N* = 6912) indicate that no current metacognitive performance measure is independent of type 1 performance. The shape of this dependency is largely reproduced by extending current models of metacognition with a source of metacognitive noise. Moreover, the reliability of metacognitive performance measures is highly sensitive to the combination of type 1 performance and trial number. Importantly, trial numbers frequently employed in metacognition research are too low to achieve an acceptable level of test-retest reliability. Among common task characteristics, simultaneous choice and confidence reports most strongly improved reliability. Finally, general recommendations about design choices and analytical remedies for studies investigating metacognitive performance are provided.

## Introduction

Being able to introspect about the correctness of thoughts and actions comes with clear benefits both at an individual and a group level. For instance, at an individual level, accurately judging confidence in one’s beliefs allows one to balance the costs and benefits of possible actions associated with these beliefs (Fleming *et al.* 2012). At a group level, communicating accurate levels of certainty optimizes collaborative decision-making (Frith 2008; Bahrami *et al.* 2012).

Researchers have long aimed to develop measures that accurately capture the performance in such metacognitive judgements. One of the earliest methods is based on the concept of ‘type 2’ receiver operating characteristic (ROC) curves (Clarke *et al.* 1959; Pollack 1959). Whereas regular ROC curves contrast the ‘objective’ probabilities of false alarms and hits, type 2 ROC curves contrast the ‘subjective’ probabilities of being correct between instances in which those decisions are factually correct (‘type 2 hit’) and incorrect (‘type 2 false alarm’). Thus, while the area under the type 1 ROC curve provides a measure for type 1 sensitivity, the area under the type 2 ROC curve (AUROC2) provides a measure of ‘metacognitive sensitivity’ (Hosseini and Ferrell 1982; Critchfield 1993; Galvin *et al.* 2003).

A key advantage of AUROC2 over more simple measures such as the correlation between accuracy and confidence (cf. Nelson 1984) is that AUROC2 is insensitive to metacognitive biases, i.e. whether an observer generally prefers lower or higher confidence ratings. However, AUROC2**—**like correlation-based measures**—**is dependent on type 1 performance. This can intuitively be understood in terms of type 1 performance influencing the proportion of trials in which an observer has to guess. As even a metacognitively ideal observer cannot predict which decisions, among guessing trials, will be correct, AUROC2 necessarily decreases with an increasing proportion of guessing trials and thus decreasing performance (for a mathematical treatment, see Galvin *et al.* 2003). Thus, AUROC2 cannot isolate metacognitive performance from type 1 performance.

A solution to this issue was proposed by Maniscalco and Lau (2012, 2014) through a measure called meta-*dʹ*. The idea of meta-*dʹ* is to express metacognitive sensitivity in terms of the type 1 sensitivity that an ‘ideal’ metacognitive observer would need in order to achieve the observed type 2 hit and false alarm rates. Since meta-*dʹ* is expressed in units of *dʹ*, it can be directly compared to**—**and normalized by**—**type 1 sensitivity. In this way, differences in metacognitive performance that are expected on the basis of type 1 performance differences alone can be mathematically corrected for. If the observer is indeed metacognitively optimal, meta-*dʹ* =  *dʹ* is expected, whereas values of meta-*dʹ* below *dʹ* indicate varying degrees of metacognitive suboptimality. As pointed out by Maniscalco and Lau (2012), due to the ratio scaling properties of *dʹ* measures, this normalization may be achieved either by subtraction (*M*_diff_ = meta-*dʹ* −  *dʹ*) or division (*M*_ratio_ = meta-*dʹ*/ *dʹ*). Fleming and Lau coined the term ‘metacognitive efficiency’ for these two measures, as they quantify how efficiently observers make use of the available type 1 information for metacognitive judgements (Fleming and Lau 2014).

Since their inception around 10 years ago, *M*_diff_, and in particular *M*_ratio_, have grown in popularity and are now in widespread use in the metacognition community. A frequent use case is between-subject designs in which performance levels might differ between participants and/or groups and which thus explicitly require a measures invariant to type 1 performance (Baird *et al.* 2014; Hauser *et al.* 2017; Sadeghi *et al.* 2017; Faivre *et al.* 2020; Hertz *et al.* 2020; Nicholson *et al.* 2020; Ordin *et al.* 2020;Reyes *et al.* 2020). Other use cases are within-subject designs in which metacognitive performance is compared between experimental conditions (Maniscalco and Lau 2015; Odegaard *et al.* 2018; Shekhar and Rahnev 2018; Filevich *et al.* 2020; Konishi *et al.* 2020; Mei *et al.* 2020; Ordin and Polyanskaya 2020) or in which relationships between two domains (e.g. brain and behaviour) are assessed (Baird *et al.* 2013, 2015; McCurdy *et al.* 2013; Fitzgerald *et al.* 2017; Samaha and Postle 2017; Lee *et al.* 2018; Ye *et al.* 2019). In both cases, difference in the underlying type 1 performance would be a confounding variable. A particular focus has been the question whether metacognitive performance is a trait-like construct that generalizes across sensory modalities or domains (e.g. memory and perception), with so far mixed results (Baird *et al.* 2013; McCurdy *et al.* 2013; Samaha and Postle 2017; Lee *et al.* 2018), pointing also to a role of task design (Samaha and Postle 2017; Lee *et al.* 2018).

Yet, despite the broad acceptance of metacognitive performance measures based on meta-*dʹ*, it is largely unknown whether the claim of type 1 performance invariance holds in practise. Indeed, a recent study demonstrated via simulation that type 1 performance invariance breaks down when assuming that confidence ratings are influenced by additional sources of (metacognitive) noise (Bang *et al.* 2019). Specifically, according to their simulation, both *M*_ratio_ and *M*_diff_ increase with increasing levels of sensory noise. To test this prediction, Bang and colleagues additionally performed a behavioural study in which sensory noise of participants was decreased through training over multiple days in a perceptual learning paradigm. Consistent with the model prediction, metacognitive efficiency decreased over the course of the experiment. Irrespective of this particular empirical finding, it seems highly likely that confidence ratings are influenced by additional sources of noise, both during the computation of metacognitive estimates and during report.

A second requirement of quantitative psychological constructs is a sufficient degree of test-retest reliability. All else equal, two measurements of metacognitive performance should give comparable results between a test and a retest session. Indeed, measurement errors for metacognitive performance are a priori expected to be rather high, as they are influenced by measurement errors of both type 1 and type 2 performance. Moreover, type 1 performance by itself is a notoriously noisy measure, as it is derived from a binary variable (correct/incorrect).

The goal of the present work is to shed light on both issues, type 1 performance independence and reliability (test-retest). My approach was two-fold in both cases. In a first step, simulated data were used to study the characteristics of metacognitive performance measures under controlled settings. Second, the same analysis used for simulation was applied to empirical data, making use of the recently released Confidence Database (Rahnev *et al.* 2020), a continuously growing collaborative repository of confidence datasets (145 at the time of accessing the database). This database comprises a large number of modalities, paradigms and various types of confidence reports and thus provides a powerful dataset to robustly assess measures of metacognition.

Overall, two measures of metacognitive sensitivity (meta-*dʹ* and AUROC2) were assessed and several variants of metacognitive efficiency: *M*_diff_, *M*_ratio_ and *M*_ratio_ with excluding extreme values, and three regularized variants of *M*_ratio_ (bounded, logarithmic and hierarchical *M*_ratio_). To assess type 1 performance invariance, sensory noise was systematically varied for the simulation-based analysis, whereas the natural variation of type 1 performance was utilized for the empirical analysis of the Confidence Database. To assess test-retest reliability, two artificial sessions of an experiment were generated for simulation-based analysis and split-half subsets of each participant’s data were created for the empirical analysis.

The paper is structured in three parts. The first and the second part are concerned with type 1 performance dependency and reliability of metacognitive performance measures, respectively. In the third part, I investigate task characteristics of studies in the Confidence Database that affect the test-retest reliability of metacognitive performance measures.

## Results

**Figure 1.**
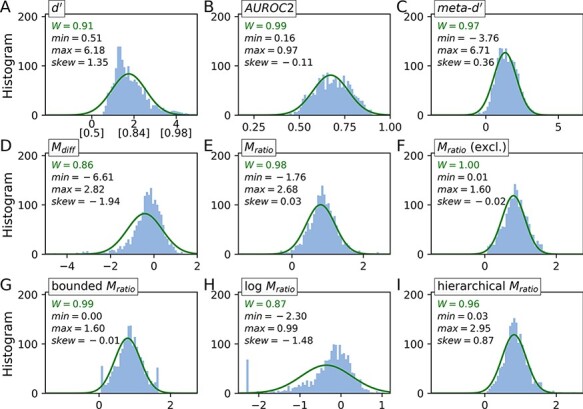
Distributions of *dʹ* and metacognitive performance measures in the Confidence Database. Only subjects with at least 400 trials were included. (A) type 1 performance measured as *dʹ*. Values in square brackets on the *x*-axis represent proportion correct responses assuming an equal proportion of trials for both stimulus categories. (B) Area under the type 2 receiver operating curve (AUROC2). (C) meta-*dʹ*. (D) *M*_diff_, the difference between meta-*dʹ* and *dʹ*. (E) *M*_ratio_, the ratio of meta-*dʹ* and *dʹ*. (F) Excluding participants with an *M*_ratio_ lower than 0 or higher than 1.6. (G) Bounded *M*_ratio_ with lower bound 0 and a symmetric upper bound of 1.6 (both the mean and the median are close to 0.8). (H) Logarithmized *M*_ratio_ in which *M*_ratio_ values are floored at 0.1 before logarithmization. Note that the normal fit is almost unaffected when excluding the subjects of the lower bound peak. (I) Hierarchical *M*_ratio_, based on Bayesian parameter estimation (Fleming 2017). Green lines indicate fits of a normal distribution (scaled by the histogram amplitude). For each distribution, the W statistic based on a Shapiro–Wilk normality test (higher values indicate higher normality; Shapiro and Wilk 1965), minimum/maximum values and the Fisher–Pearson coefficient of skewness are provided

Figure 1 introduces the type 1 and type 2 performance measures investigated in this study in terms of their distribution in the Confidence Database. For the creation of these distributions, I included participants with at least 400 trials and a certain level of above-chance performance (*dʹ* > 0.5).

Type 1 performance *dʹ* shows a peak at an intermediate performance level (corresponding to around 75% correct responses), which is likely due to staircase procedures that often target intermediate performance levels. The distribution has a positive skew. While the distributions of the two metacognitive sensitivity measures, AUROC2 and meta-*dʹ*, are relatively symmetric and close to a normal distribution, the histogram of *M*_diff_ is clearly affected by type 1 performance (the asymmetry is in the opposite direction to *dʹ*, as *M*_diff_ involves a subtraction of *dʹ*).

By contrast, the distribution of *M*_ratio_ is closer to a normal distribution. However, despite the conservative inclusion criteria, *M*_ratio_ shows a significant fraction of unrealistically extreme values. 2.4% of subjects have negative *M*_ratio_ values and 4% have *M*_ratio_ values higher than 1.5. This problem is more severe in studies with fewer trials per subject (e.g. 4% negative values and 10% values >1.5 in studies with less than 200 trials).

To address the instability of *M*_ratio_, we additionally evaluated a scenario in which extreme values of *M*_ratio_ are excluded, as well as three regularization methods. Figure 1F shows the distribution of *M*_ratio_ values after excluding participants with negative *M*_ratio_ values (affecting 2.4% of the *M*_ratio_ values in Fig. 1E) and *M*_ratio_ values higher than an upper bound of 1.6 that is symmetric with respect to the median of 0.8 (affecting likewise 2.4% of *M*_ratio_ values). This measure is henceforth referred to as *M*_ratio_ (excl.). As a result of excluding these participants, the normality of the *M*_ratio_ distribution slightly increases.

The first regularization method, ‘bounding’, forces lower and upper bounds for *M*_ratio_. To my knowledge, this method has not yet been used in the literature. In the absence of a reference, I thus chose the same bounds of 0 and 1.6 as discussed above, which introduces a noticeable probability mass at both bounds (Fig. 1G). A second regularization method, taking the ‘logarithm’, was suggested by Fleming and Lau (2014) to address the occurrence of extreme values and non-normality. However, as Fig. 1H shows, taking the logarithm leads to a heavily asymmetric distribution with a long left tail. If the goal is normality, taking the logarithm is thus not advised. Finally, I tested a ‘hierarchical’ Bayesian estimation method introduced by Fleming (2017) that effectively regularizes extreme values by means of a group prior. The distribution of hierarchical *M*_ratio_ values shows a slight positive skew (Fig. 1I).

### The relationship between metacognitive performance measures and type 1 performance

#### Simulation

In a first step, I simulated the relationship between the investigated set of metacognitive performance measures and type 1 performance. The advantages of simulated data are that they allow precise control over underlying parameters of the generative model, a systematic evaluation across an arbitrary range of relevant variables (including sample size) and high statistical power. At the same time it must be kept in mind that simulated models rely on assumptions about the process of data generation that may deviate from the empirical truth.

Here, I assume that confidence is computed based on the subjective probability of being correct, i.e. using the assumption that observers have a reliable estimate of the stimulus-generating process (in particular an estimate of their own sensory noise). Equipped with such an estimate and using Bayes’ rule, an observer is then able to compute a choice probability (see section ‘General model’; Equation 3) and can use it to compute confidence (Equation 4). I assume that reported levels of confidence are subject to metacognitive noise, described by a Beta distribution (Equation 6). The Beta distribution lends itself for this purpose as it is, by design, a distribution that characterizes the uncertainty of probability estimates. It is thus bounded between 0 and 1 and implicitly avoids other nonsensical values. It can be parameterized with a spread parameter *σ*_m_, which henceforth is referred to as the metacognitive noise parameter. The lower the *σ*_m_, the more precise the reported confidence will reflect the choice probability. For the maximum value of *σ*_m_ = 0.5, the Beta distribution approaches the uniform distribution and thus confidence reports become random uniform draws from the interval [0; 1].

**Figure 2.**
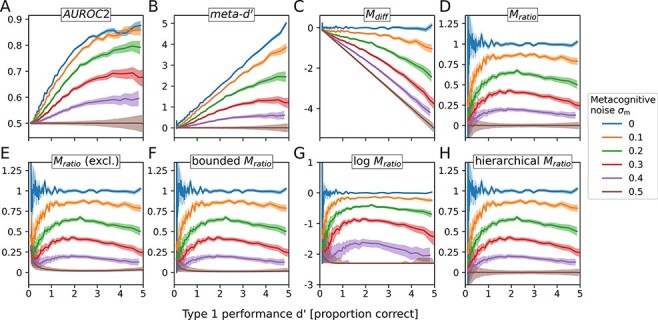
Simulation: relationship between type 1 (*dʹ*) and type 2 performance. Shaded areas denote asymmetrical standard deviations. (A–B) Both measures of metacognitive sensitivity**—**AUROC2 and meta-*dʹ***—**increase with increasing type 1 performance. (C) *M*_diff_ decreases with increasing type 1 performance and more strongly so for increasing levels of metacognitive noise. (D) *M*_ratio_ is more stable across different performance levels compared to *M*_diff_ but is likewise not independent of type 1 performance in the simulation. An initial pronounced increase at low levels of performance is followed by a slight decrease starting from *dʹ* values of around 2 (corresponding to a performance of around 80% correct). (E) Excluding participants with an *M*_ratio_ lower than 0 or higher than 1.6. (F–H) In the simulation, regularization of *M*_ratio_ has no noticeable effect on its dependency on type 1 performance

For the purpose of describing the relationship between metacognitive performance and type 1 performance, I simulated data for varying levels of sensory and metacognitive noise. As expected, the two measures of metacognitive sensitivity, AUROC2 (Fig. 2A) and meta-*dʹ*, (Fig. 2B) increase with increasing type 1 performance despite constant metacognitive noise.

Measures of metacognitive ‘efficiency’, on the other hand, claim to be invariant with respect to type 1 performance. In the case of *M*_diff_, which corresponds to the subtraction of meta-*dʹ* and *dʹ*, this is not the case. As shown in Fig. 2C, *M*_diff_ shows a clear negative relationship with increasing type 1 performance with the slope becoming more negative with increasing levels of metacognitive noise. This can be understood with an extreme example. Consider an observer that has very high metacognitive noise such that they essentially pick confidence ratings at random. Clearly, for this observer, meta-*dʹ* should be equal or close to zero irrespective of type 1 performance. If one thus subtracts type 1 performance (*dʹ*) from meta-*dʹ*, the observed negative relationship is expected. Lower levels of metacognitive noise only attenuate this negative relationship, but even at moderate levels this bias is still substantial.

By contrast, *M*_ratio_ is largely stable across different type 1 performance levels (Fig. 2D). While for *M*_diff_, the bias is worst for the highest level of metacognitive noise, *M*_ratio_ is most unstable for intermediate values of metacognitive noise. In the case of purely random confidence ratings, *M*_ratio_ stays flat at zero (as it should), simply because the nominator meta-*dʹ* is zero. Also for the other extreme**—**metacognitive noise close to zero**—***M*_ratio_ yields a stable estimate of 1. However, for intermediate values of metacognitive noise, *M*_ratio_ drops to zero as type 1 performance approaches chance level (*dʹ* = 0) and also shows a slightly negative slope at higher levels of type 1 performance. The general pattern is very similar when excluding participants with extreme *M*_ratio_ values below 0 or above 1.6 (Fig. 2E), or for regularized variants of *M*_ratio_ (Fig. 2F–H). A minor deviation can be observed for *M*_ratio_ (excl.), where a slight positive bias is visible at low levels of type 1 performance and high levels of metacognitive noise.

Overall, while the type 1 performance dependence of AUROC2 and meta-*dʹ* is well-known and expected, the simulation analyses suggest that the putatively performance-independent measures *M*_diff_ and *M*_ratio_ do not necessarily hold up to their claim. The performance dependency is particularly clear for *M*_diff_, which should not be used as a measure of metacognitive efficiency. The performance dependency of *M*_ratio_ is weaker and more complex, depending on the expected range of both type 1 and type 2 performance.

#### Empirical data

In a next step, I evaluated the type 1 performance dependency of metacognitive efficiency measures on empirical data of the Confidence Database, which provides high statistical sample power across a variety of tasks and modalities (Rahnev *et al.* 2020). I did not test the two measures of metacognitive sensitivity, AUROC2 and meta-*dʹ*, as they are a priori expected to be dependent on type 1 performance (also confirmed in the simulation).

To test for a linear relationship between type 1 performance and each metacognitive efficiency measure, mixed linear models were used with study as a grouping variable and modality (cognitive, memory, motor perception, mixed) as an additional control variable. Moreover, each participants’ data were split into two interleaved halves, such that the metacognitive performance measures were computed on a different subset of the data than the type 1 performance measure *dʹ*. This ensured that the measurement noise associated with the estimation of *dʹ* per se and the measurement noise of the *dʹ* that enters the computation of metacognitive efficiency measures (either via subtraction or division) were independent, thus preventing spurious correlations.

**Figure 3.**
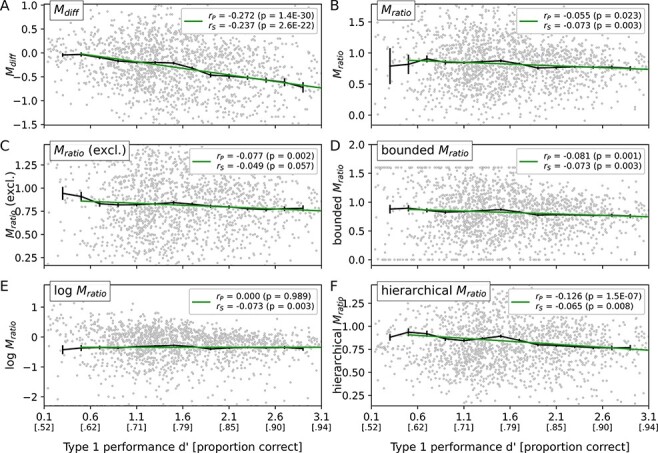
Relationship between type 1 performance (*dʹ*) and metacognitive efficiency measures in the Confidence Database. (A) Like in the simulation, *M*_diff_ shows a clear negative relationship with *dʹ*. (B) *M*_ratio_ likewise shows a similar pattern as the simulation: a slight initial increase is followed by a decrease with increasing *dʹ*. (C) Excluding participants with *M*_ratio_ values lower than 0 or higher than 1.6. (D–F) Regularized variants of *M*_ratio_: bounding, logarithmic transformation and hierarchical estimation (Fleming 2017). For all methods except the bounded *M*_ratio_, the range of the *y* axis was restricted to the 95% of data points, which were closest to the overall median. Black error bars indicate the mean and the standard error of mean (SEM) of bins centred at the position of the errorbar and extending Δ*dʹ* ± 0.2 left and right of the centre. Green lines show the linear regression line to all data points that match the inclusion criterion of *dʹ* > 0.5

In agreement with the simulation, *M*_diff_ shows a strong negative relationship with type 1 performance (beta ± SEM = −0.17 ± 0.02, *P* < 0.001; Fig. 3A). Thus, also empirically *M*_diff_ is not independent of type 1 performance and should thus not be used as a measure of metacognitive efficiency.

*M*_ratio_ (without regularization) likewise shows a similar pattern as in the simulation. In particular, *M*_ratio_ shows a slight but steady decrease with increasing type 1 performance (beta = −0.08 ± 0.03, *P* = 0.010). As in the simulation, bounding and exclusion of extreme *M*_ratio_ values had only moderate effects on the type 1 performance dependency. In both cases, the negative relationship with *dʹ* was numerically weaker, but still trendwise significant (bounded *M*_ratio_: beta = −0.07 ± 0.04, *P* = 0.063; *M*_ratio_ with exclusion: beta = −0.08 ± 0.04, *P* = 0.072). Of note, when pooling participants across studies in a simple regression analysis, the statistical evidence for this negative relationship was stronger (cf. Fig. 3C and D).

By contrast, the hierarchical estimation and the logarithmization of *M*_ratio_ showed an effect with respect to the type 1 performance dependency. While the negative relationship was even more pronounced for the hierarchical *M*_ratio_ (beta = −0.19 ± 0.05, *P* < 0.001; Fig. 3F), compared to the regular *M*_ratio_, it disappeared for the logarithmized *M*_ratio_ (beta = 0.01 ± 0.02, *P* = 0.581). Of note, when pooling across participants, there was still a negative rank-order correlation between the logarithmized *M*_ratio_ and *dʹ* (*r*_s_ = −0.073, *P* = 0.003;  Fig. 3E). More data are thus necessary to conclude with certainty that the relationship is indeed absent.

While the empirical data thus largely reproduced the decrease of *M*_ratio_ with higher type 1 performance in the simulation, the initial increase of *M*_ratio_ at low levels of type 1 performance is much less evident. The regular *M*_ratio_ shows a tendency in this direction (Fig. 1B), but the scarcity of participants with very low type 1 performance levels prevents a meaningful statistical analysis of this effect. When excluding *M*_ratio_ values with extreme values (Fig. 1C), this tendency disappears entirely; however, this might be due to the positive bias at low type 1 performance levels observed in the simulation (cf. Fig. 2E).

By and large, the results based on the Confidence Database confirm the results of the simulation: a strong negative type 1 performance dependency for *M*_diff_ and a weaker, possibly more complex dependency of *M*_ratio_ and its regularized variants, with a general trend towards lower *M*_ratio_ values with higher *dʹ*.

### Test-retest reliability of metacognitive performance measures

#### Simulation

Test-retest reliability is a critical hallmark of any psychological construct and little to nothing is currently known about the reliability of metacognitive performance measures. As for the type 1 performance dependency, I first set out to evaluate the reliability of these measures under the controlled settings of a simulation.

To this aim, I systematically varied the number of trials per subject and the average performance level. For each number of trials and performance level, two artificial experimental sessions were simulated for 100 subjects, referred to as ‘test’ and ‘retest’ session, which allowed quantifying the test-retest reliability. Metacognitive noise of each subject was drawn from a uniform distribution covering the entire range from an ideal metacognitive observer (*σ*_m_ = 0) to a metacognitively blind observer (*σ*_m_ = 0.5), which effectively chooses confidence ratings at random.

Two measures of reliability were employed: Pearson correlation and the normalized mean absolute error (NMAE). Whereas the Pearson correlation indicates whether the pattern of results across participants is similar between two measurements, the NMAE provides information about absolute measurement errors. To allow a comparison between metacognitive performance measures, the NMAE normalizes the mean absolute error between the test and retest session values by the mean absolute error between each individual value and the ‘average’ in the other session. An NMAE of e.g. 0.5 thus indicates that the average difference between test and retest values is half of the average distance between the values and the average of the other session.

For the reliability analysis, I focus on the *M*_ratio_ and its regularized variants, as all other metacognitive performance measures are strongly dependent on type 1 performance. This makes it impossible to dissociate whether the reliability is driven by the consistency of type 1 or type 2 performance. For completeness, the reliability of other metacognitive performance measures as well as type 1 performance is provided in Supplementary Figure S1.

**Figure 4.**
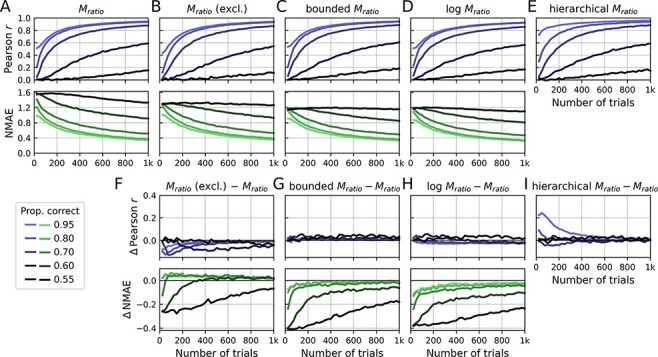
Simulation: test-retest reliability of metacognitive performance measures. For each number of trials and performance level, two hypothetical experimental sessions of 100 subjects were simulated, corresponding to test and retest. This procedure was repeated for *X* iterations and averages were computed across iterations. Metacognitive performance measures [*M*_ratio_, *M*_ratio_ (excl.), bounded *M*_ratio_, log *M*_ratio_ and hierarchical *M*_ratio_] were computed for each session separately. Reliability was quantified either by means of the Pearson correlation or the NMAE. Note that the NMAE is not valid for the hierarchical *M*_ratio_ (see section ‘Measures of test-retest reliability’) and is thus not shown. (A–E) Pearson correlation and the NMAE quantify the reliability of metacognitive performance measures between test and retest. Note that exceedingly extreme values of the regular *M*_ratio_ (| *M*_ratio_| > 10) were excluded, as they caused some instability in the analysis. (F–I) Differences in test-retest reliability between measures

As expected, the reliability of *M*_ratio_ increases with the number of trials per subject, i.e. increasing correlation and decreasing NMAE (Fig. 4). Likewise, the reliability increases with increasing type 1 performance. Intuitively, this is because the precision of metacognitive performance estimates naturally increases as the proportion of guessing trials decreases. In contrast, as type 1 performance approaches the chance level, there is a dramatic drop in reliability. For instance, at 250 trials and 60% correct responses, the Pearson reliability is at only *r* = 0.2.

To put the overall level of reliability into context, it is worth contrasting it with the reliability of type 1 performance. The Pearson reliability of type 1 performance is close to 1 already at around 250 trials (Supplementary Figure S1A)—despite the fact that it is based on noisy binary values (correct/incorrect). In comparison, the reliability of *M*_ratio_ is substantially lower in this trial range, not least due to the fact that it is based on a combination of two noisy variables—type 1 and type 2 responses.

Excluding extreme values of *M*_ratio_ (Fig. 4B and F) has mainly an effect on the NMAE at low performance levels and low trial numbers. This is not surprising given the fact that these are the conditions that typically produce extreme *M*_ratio_ values. Of note, the Pearson correlation appears to be minimally higher for the *M*_ratio_ without exclusion, at least under some conditions. A potential explanation is that extreme values introduce larger variance, which might have a slight net positive effect on the Pearson correlation.

Can regularization improve the reliability? Taking the logarithm of the *M*_ratio_ improves the Pearson reliability only for lower levels of type 1 performance or at lower trial numbers and thus only when *M*_ratio_ is expected to be unstable. At typical performance levels (>70% correct) and trial numbers, the simulation suggests that test-retest reliability can even be worse than the original *M*_ratio_. By contrast, bounding values of *M*_ratio_ improves the Pearson correlation across simulation parameters. Again, improvements are largest at lower levels of type 1 performance and at a lower number of trials. This analysis suggests that—in terms of test-retest reliability—bounding should be preferred over taking the logarithm (see also Supplementary Figure S2, for a direct comparison of regularization/exclusion methods). Finally, the reliability of the hierarchical *M*_ratio_ is highly similar to the bounded *M*_ratio_ with the exception of high type 1 performance levels at low trial numbers: here, the hierarchical *M*_ratio_ clearly outperforms all other measures.

Overall, I conclude that the reliability of *M*_ratio_ will be poor in many realistic scenarios. Studies investigating metacognitive efficiency should thus carefully consider the number of trials and the targeted type 1 performance level, both of which are factors that substantially affect reliability. Regularization methods improve the reliability to a certain degree and are thus generally recommended. Between regularization methods, the simulation shows comparable improvements of the bounded and hierarchical *M*_ratio_ in terms of Pearson reliability, while the logarithmic *M*_ratio_ performs worse and can even lead to a decrease in Pearson reliability.

#### Empirical data

To assess the test-retest reliability of metacognitive performance measures on empirical data, the data of each subject were divided in two artificial subsets through an interleaved splitting procedure. As the simulation indicated that the trial number per subject is a critical quantity for the reliability of metacognitive performance measures, the studies of the Confidence Database were binned according to the split-half number of trials per subject (1st bin: 0–200 trials, … 5th bin: 800–1000 trials). Since there was some variance of trial numbers within studies, I excluded participants whose trial numbers were outside the assigned bin of a study. As the large majority of studies have intermediate performance levels (85% of the studies have an average proportion correct between 0.65 and 0.85), the studies were not split up according to performance. Importantly, the performance is quite similar for all trial number bins (Fig. 3A, black line). As in the simulation, the focus was on *M*_ratio_ and its regularized variants (for other measures see Supplementary Figure S3).

Without regularization and at moderate trial numbers (<400), the Pearson reliability of *M*_ratio_ is quite poor at *r* ≤ 0.6 and the NMAE is ≥1, indicating that the *M*_ratio_ of a subject at test is, on average, not closer to the same subject’s *M*_ratio_ at retest than it is to the mean of all subjects (Fig. 3A). The Pearson correlation increases to around 0.7 and 0.85 for trial numbers between 400–600 and 600–800, respectively, and the NMAE drops below 1. Overall, 400 appears as a sensible minimum recommended trial number for studies using the regular *M*_ratio_.

**Figure 5.**
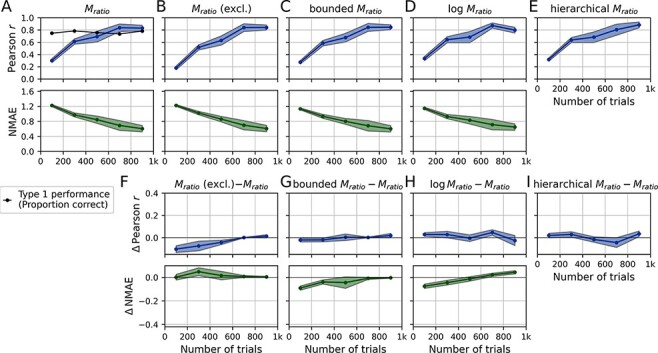
Test-retest reliability of metacognitive performance measures in the Confidence database. Test-retest reliability is computed either as the Pearson correlation coefficient or the NMAE between test and retest trials. The *x*-axis denotes the average split-half number of trials for the Confidence Database studies (bins of 200 from 0 to 1000 trials), i.e. the number of trials per test and retest. In this way, the expected reliability for other/new studies with overall *N* trials can simply be obtained by looking up the reliability for *x* = *N*. The accuracy (proportion correct) is relatively constant across bins and thus likely not a confounder. Note that the number of samples refers to the split-half test and retest datasets; hence, the total number of samples per subject is twice as high. The shaded areas indicate standard errors across studies. (A–E) Test-retest reliability of *M*_ratio_, *M*_ratio_ (excl.) and regularized variants. For convenience, type 1 performance is plotted in panel A, given as proportion correct responses. Note that the NMAE is not valid for the hierarchical *M*_ratio_ (see section ‘Measures of test-retest reliability’) and is thus not shown. (F–I) Differences in test-retest reliability between *M*_ratio_ and *M*_ratio_ (excl.)/regularized variants of *M*_ratio_

Does regularization improve the reliability of *M*_ratio_? At low to moderate trial numbers, bounding and logarithmization of *M*_ratio_ values lead to slight improvements over the regular *M*_ratio_ in terms of NMAE reliability (Fig. 3B–G). The Pearson reliability is largely unaffected by regularization and also does not exhibit the slight advantage of the hierarchical *M*_ratio_ observed for high type 1 performance levels in the simulation. There are no detectable differences between the regularization methods (more data would be necessary). Similar to the simulation, excluding *M*_ratio_ values slightly decreased the test-retest reliability (Fig. 5F, upper panel). An advantage in terms of NMAE reliability was not observed (Fig. 5F, lower panel).

As to be expected, the benefit of regularization vanishes entirely at larger trial numbers. For studies with around 600 trials or more, there is no longer an improvement of NMAE reliability for any regularization method. This suggests that studies that are powered with 600 trials and more per subject can quite safely omit regularization.

### Task characteristics affecting the reliability of metacognitive performance measures

As shown in the previous section, the number of trials and type 1 performance constrain the expected reliability of *M*_ratio_. Both factors must be carefully chosen to achieve an acceptable level of measurement reliability. In this final section I was interested in which other study and task characteristics affect reliability of measured *M*_ratio_ values. Specifically, I was interested in the following five task characteristics: (i) the number of available confidence ratings; (ii) whether ratings are continuous or not; (iii) whether confidence ratings are provided simultaneously with type 1 choices; (iv) whether feedback is provided and (v) whether there is an online staircase procedure. I focused on the reliability of regular *M*_ratio_ without regularization and therefore selected only studies with at least 400 trials, in line with the recommendations of the previous sections.

The test-retest reliability of *M*_ratio_ was computed separately for each study and defined a mixed linear model with the test-retest reliability as the dependent variable and the above task characteristics as the independent variables of interest. Additional control variables were the number of subjects, the number of trials and study-specific averages of type 1 performance (*dʹ*), confidence, *M*_ratio_ and modality (categorical predictor: cognitive, motor, perception, mixed or memory). The grouping variable of the mixed model was ‘study id’, i.e. a unique identifier for each study.

**Figure 6.**
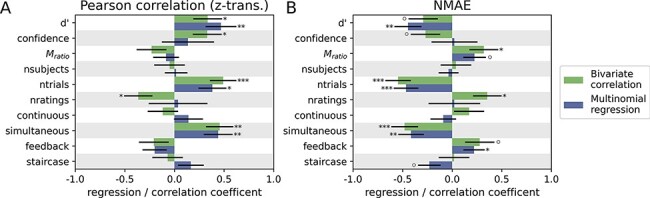
Study characteristics affecting the test-retest reliability of *M*_ratio_. Green bars indicate the bivariate correlation coefficient between the predictor and the measure of reliability; blue bars show the regression coefficient of a linear model including all predictors. (A) Test-retest reliability measured with the Pearson correlation coefficient. Positive values indicate improvements in reliability. (B) Test-retest reliability measured with the NMAE. Negative values indicate improvements in reliability. °*P* < 0.1, *P < 0.05, **P < 0.01, ***P < 0.001

Confirming the previous analyses, type 1 performance and the number of trials strongly predict the test-retest reliability of *M*_ratio_ (Fig. 6). Among the variables of interest, simultaneous choice and confidence responses and the number of available ratings were associated with higher reliability (only bivariate correlation) and, somewhat surprisingly, external feedback was associated with slightly lower reliability.

The positive effect of simultaneous choice and confidence ratings on the reliability of *M*_ratio_ was strong. While the average test-retest correlation is *r* = 0.83 when responses are provided simultaneously, it drops to *r* = 0.51 when confidence ratings are provided in a separate response after the type 1 choice. Online staircasing showed a trendwise association with lower reliability, which may be interesting in reference to the ongoing discussion about the interaction of staircase procedures and *M*_ratio_ (Rahnev and Fleming 2019).

It is notable that test-retest reliability was unaffected by whether confidence ratings were given on a discrete or continuous scale, although a higher number of available confidence levels slightly improved the Pearson reliability.

The strongest and most robust conclusion from this analysis is the clear benefit of simultaneous type 1 and type 2 responses on the test-retest reliability of *M*_ratio_.

## Discussion

In the current work, I investigated the type 1 performance independence and reliability of metacognitive performance measures. I showed that even measures of metacognitive *efficiency*, i.e. measures that normalize for type 1 performance, are not necessarily independent of type 1 performance. This dependency is much weaker and thus possibly tolerable for *M*_ratio_ compared to *M*_diff_. The analyses of test-retest reliability showed that *M*_ratio_ has quite poor test-retest reliability and standard trial numbers; moreover, the reliability analyses provide guidance and recommendations for regularization methods, trial numbers and task design.

### All current measures of metacognitive performance are dependent on type 1 performance

A quintessential aspect for measures of metacognitive performance is to what degree a measure isolates type 2 from type 1 performance. The intricacy of the tight relationship between type 1 and type 2 has been long known and has led to the development of meta-*dʹ* (Rounis *et al.* 2010; Maniscalco and Lau 2012, 2014) and two proposals for metacognitive performance measures that explicitly normalize for type 1 performance (*M*_diff_ and *M*_ratio_). Here, I showed through simulation and application to empirical data that *M*_diff_ is heavily biased by type 1 performance and thus should not be used if independence of type 1 performance is important. Specifically, *M*_diff_ decreases with increasing type 1 performance and this negative relationship becomes stronger with increasing metacognitive noise.

While *M*_ratio_ is clearly more stable across different levels of type 1 performance, it may not be entirely independent. Also here, simulation and application to empirical data show agreement and indicate a relationship between type 1 performance and *M*_ratio_ that can be described as an inverted U-shape: as type 1 performance approaches the chance level, *M*_ratio_ approaches zero; with increasing type 1 performance, *M*_ratio_ shows a slight but a steady decrease. Of note, this pattern is independent of the number of trials: the simulation used a very large number of 10 000 trials and the pattern did not change when the number of trials was either increased or decreased.

A limitation of simulation-based analyses is that any result could be conditional on the chosen model and the relationship between *M*_ratio_ and type 1 performance may be of a different kind for the unknown ground truth model. While it is a good sign that the simulation reproduced the general pattern of the empirical data, it is not a proof. This is less of an issue for the conclusions about *M*_diff_, since its type 1 performance dependency can be logically explained and its empirical negative relationship with type 1 performance was so strong that a confirmation by simulation is less important. However, for *M*_ratio_ neither of these arguments can be used. In an exploratory analysis I therefore probed the robustness of the simulation result for *M*_ratio_ by testing additional metacognitive noise distributions. As shown in Supplementary Figure S4, the results were highly similar to the Beta distribution used in the main simulation. Future studies could test the type 1 performance dependency for different model architectures as well.

An additional aspect to consider is that there might be a true relationship between type 1 performance and metacognitive ability, which is not due to a measurement confound. For instance, there could be a true positive relationship reflecting the general observation that cognitive abilities correlate across domains (Carroll 1993). In this view, the observed negative correlation of the measures might factually be stronger and is masked to some degree by the general positive relationship in the population. Support for this view comes from a recent large-scale model-based analysis of the Dunning–Kruger effect, which concluded that low performers (in the type 1 task) also tend to have more noisy representations of their type 1 accuracy (Jansen *et al.* 2021; see also Mazor and Fleming 2021). Alternatively, there could be a true negative relationship because of a potential trade-off in allocating cognitive resources to either the type 1 or the type 2 task. While this highlights the difficulty to conclusively prove the ‘non-independence’ of metacognitive performance measures and type 1 performance, the burden of proof certainly lies on the side of those claiming ‘independence’.

### Possible solutions to address the dependency on type 1 performance

While the type 1 performance dependency of *M*_ratio_ may be regarded as relatively subtle, it can nevertheless be an issue, especially as higher and higher sample sizes are collected in online experiments or collaborative research projects. Large sample sizes will be sensitive even to small type 1 performance confounds and lead to artifactual results.

An obvious solution to this issue is online staircase procedures that continuously adjust stimulus difficulty towards a constant target type 1 performance level (e.g. Rouault *et al.* 2018). However, staircase procedures introduce a different issue. As noted by Rahnev and Fleming (2019), a mix of stimulus difficulty levels can artificially inflate estimates of metacognitive performance relative to a design with a single difficulty level. In brief, the issue is that it is easier for subjects to assign high confidence to correct choices and low confidence to incorrect choices when the experiment itself contains objectively easier and harder trials. This might not be a problem, if the staircase-induced inflation would be a constant shift that cancels out in a comparison between conditions. However, there is no guarantee that the mix of easier and hard trials is identical for different subjects and thus also no guarantee that the shift is constant. For instance, relative to control subjects, a group of patients might show stronger fluctuations of concentration, which could lead to larger adjustments in the staircase procedure. Larger differences in objective stimulus difficulty in turn could lead to an inflated estimate of metacognitive performance. For this reason, Rahnev and Fleming (2019) generally recommend a single-difficulty level, which however leads back to the problem of type 1 performance confounds. Offline staircase procedures can alleviate the issue to a certain degree. However, because offline staircase procedures are notoriously imprecise (García-Pérez 1998) and learning will often continue throughout the main experiment (not necessarily the same in different groups), it is often hard to equate performance reliably with offline staircase procedures.

At present, there is thus no all-purpose solution. In general I recommend including confounding factors in any analysis of metacognitive performance, i.e. type 1 performance in single-difficulty-level designs and staircase variability (Rahnev and Fleming 2019) in designs with an online staircase. A specific issue when controlling for type 1 performance is that *dʹ* effectively would enter both sides of the regression equation, as it is also contained in *M*_ratio_ = meta-*dʹ*/*dʹ*. In this case I recommend using a cross-validated regression approach, such that *dʹ* in the DV and IV are computed on independent data (as in the analysis in Fig. 3). While doing so, one should be aware of the fact that controlling for type 1 performance might remove true interindividual variability in metacognitive performance when type 1 performance and metacognitive ability factually correlate in the population (as noted above).

### The test**-**retest reliability of *M*_ratio_ is strongly affected by type 1 performance and the number of trials

Most studies in metacognition research aim for intermediate type 1 performance levels to introduce a sufficient degree of variance in confidence ratings. The simulation analyses showed that, in terms of test-retest reliability, it makes all the difference whether type 1 performance is at 60%, 70% or 80% correct responses. At 60% correct responses or below, the Pearson reliability of *M*_ratio_ is very poor even at decent trial numbers of around 400–600 (*r* ≈ 0.4). By contrast, at 80% correct responses the Pearson reliability is around *r* ≈ 0.8 for 400–600 trials and thus already substantially better. In the absence of other constraints, I thus recommend a performance level of approximately 80% correct responses for studies investigating metacognitive efficiency.

As for any noisy measure, the test-retest reliability of *M*_ratio_ is affected by the number of trials measured for a subject. In general, the number of trials should be higher for lower levels of type 1 performance. For instance, if type 1 performance is at 60% correct responses around 1000 trials are required according to the simulations to even achieve a Pearson reliability of *r* = 0.6. The analysis of the Confidence database, which provides a cross section of trial numbers used by studies in the research field, suggests a minimum recommended trial number of 400 trials for studies measuring metacognitive performance.

### Regularization can boost the test**-**retest reliability of *M*_ratio_

To address the instability of *M*_ratio_, I evaluated three different methods of regularization (bounded, logarithmic and hierarchical *M*_ratio_) and tested their effects on test-retest reliability. With the exception of the logarithmic *M*_ratio_, all regularization methods consistently improved the test-retest reliability. In general, regularization improved the NMAE much stronger than the Pearson correlation. This is not surprising as the NMAE is naturally improved when extreme values are tamed by regularization. Nevertheless, regularization also introduced a slight but significant advantage with respect to the Pearson reliability in both simulated and empirical data. The benefits of regularization vanished as the number of trials increased. For studies with 600 trials or more in the Confidence Database, the reliability was almost indistinguishable for *M*_ratio_ with and without regularization.

For studies measuring below 600 trials per subject, I recommend using some form of regularization. Given the heavily asymmetric distribution of *M*_ratio_ values following a logarithmic transformation, I advise against this method.

The hierarchical *M*_ratio_ can likewise be regarded as a form of regularization and performed well in terms of test-retest reliability with a pronounced advantage relative to other regularization methods for high type 1 performance levels. Nevertheless, its hierarchical estimation approach is not without problems. Hierarchical *M*_ratio_ values cannot be compared between studies (when fitted separately) as they depend on the study-specific group average. In within-subject designs, the correlation/variance structure has to be specified with great care to enable valid inferences. For instance, to my knowledge there is currently no available solution for mixed designs with both within-subject and between-subject factors. However, it should be noted that I used the hierarchical *M*_ratio_ as a means to regularize single-subject estimates, whereas its main original aim was allowing for an accurate inference on group-level parameters when individual trial numbers are limited. Although group-level measures are beyond the scope of this, given their increased popularity (e.g. Harrison *et al.* 2020), future studies should assess the reliability of these measures as well.

In terms of regularization I thus recommend a simple bounding method along the lines suggested in this work. While this method naturally introduces edge densities in the resulting distributions, these edge cases should be relatively rare for reasonable choices of type 1 performance and the number of trials. Applied to the data of the Confidence database, the distribution resulting from bounding showed a high degree of normality for the bounds applied (lower bound 0 and upper bound 1.6). The advantage compared to functional transformations such as the logarithmic transformation is that the absolute *M*_ratio_ values resulting from bounding are still in the original scale and thus interpretable.

### Task characteristics that improve the test**-**retest reliability of *M*_ratio_

I argue that the test-retest reliability of metacognitive performance measures can be used as a quality measure for the confidence ratings obtained in a study. In brief, the rationale is that more precise confidence ratings will improve the measurement reliability of the psychological construct that is computed on the basis of these ratings (i.e. metacognitive performance/ability).

Among the five task characteristics of interest (continuous versus discrete rating scales, number of discrete confidence ratings, simultaneous versus sequential type 1/2 responses, feedback, presence of an online staircase), simultaneous type 1/2 responses most strongly benefited the test-retest reliability of *M*_ratio_. Compared to post-decisional ratings of confidence, simultaneous type 1/2 responses may be more precise as they are closer to the percept and thus a potential post-decisional memory loss or other sources of noise are minimized. Nevertheless, it is worth noting that one could have made an argument for the reverse finding as well, such that sequential type 1/2 responses give more room for post-stimulus computations of confidence that ultimately lead to more precise and consistent confidence ratings.

Interestingly, contrary to the expectation, there was a weak *negative* effect of external feedback, i.e. the presence of feedback reduced the test-retest reliability of *M*_ratio_. It might be possible that feedback leads to ongoing noisy recalibrations of confidence ratings, which introduces additional variability. However, given the uncertainty of the effect, this finding should be considered as exploratory.

I conclude that studies investigating metacognitive performance should consider the use of simultaneous type 1 and type 2 responses to improve measurement reliability.

### Future directions

What may be ultimately required to isolate type 1 and type 2 performance is a mechanistic model of metacognition that captures relevant sources of metacognitive noise and biases and describes the functional transformations underlying human reports of confidence. Such a model may allow to reverse-engineer metacognitive noise parameters that characterize metacognitive ability in humans (or different facets thereof) separately from sensory or decisional noise parameters. While there are some initial proposals for such models (Bang *et al.* 2019; Shekhar and Rahnev 2021), there is currently no established model.

## Conclusion

In sum, this article investigated the type 1 performance dependency and test-retest reliability of metacognitive performance measures. It characterizes the behaviour of metacognitive performance measures in dependence of key variables (type 1 performance, overall level of metacognitive noise and number of trials) both on simulated and empirical data. On the basis of these results, it provides guidance and recommendations for researchers interested in investigating metacognitive performance.

## Methods and materials

### Empirical studies

Analyses of empirical data are based on studies of the Confidence Database, a publicly available repository of dataset with confidence ratings (Rahnev *et al.* 2020). At the time of access, the database had overall 145 studies. Included studies cover different cognitive domains (e.g. perception, memory or decision making), different confidence scales (such as binary, n-point scales, continuous scales and wagering) collect confidence at different times (for example, after or simultaneous with the decision). Inclusion criteria for studies were tasks with exactly two response options (i.e. excluding studies with more than two or continuous type 1 responses). If not otherwise specified, we included only participants with at least 400 trials and *dʹ* > 0.5, which reduced the sample size from *N* = 6912 to 1757 in these instances.

### Metacognitive performance measures

I evaluated two measures of metacognitive sensitivity (AUROC2 and meta-*dʹ*) and two measures of metacognitive efficiency (*M*_ratio_ and *M*_diff_). meta-*dʹ* and its derivatives *M*_ratio_ and *M*_diff_ were computed on the basis of Python adaptations of the original code as described in Maniscalco and Lau (2012, 2014), available at http://www.columbia.edu/∼bsm2105/type2sdt/. All codes are made available upon the final publication of this article.

Due to the instability of *M*_ratio_ at low trial numbers or low levels of type 1 performance (Fleming and Lau 2014), I evaluated a scenario in which extreme *M*_ratio_ values are excluded and three additional regularized variants of *M*_ratio_ (Table 1). In the first case, referred to as *M*_ratio_ (excl.), I excluded participants below and above specified upper bounds. A sensible lower bound is 0, which corresponds to a metacognitively blind observer. Also note that negative values of *M*_ratio_ could show nonsensical behaviour. For instance, when meta-*dʹ* is negative, *M*_ratio_ will increase with increasing (positive) type 1 performance, although it should decrease in the logic of a metacognitive performance measure. For the upper bound one might consider 1, which corresponds to an ideal metacognitive observer. However, this will often lead to an asymmetric highly non-normal distribution, as *M*_ratio_ values greater than 1 are quite common, possibly also due to post-decisional processing that benefits confidence reports. Instead, here I propose to use an upper bound that is symmetric with respect to the mean or median of *M*_ratio_ in the Confidence Database (both are around 0.8). In this work I thus use an upper bound of 1.6.

**Table 1.** Variants of *M*_ratio_

Method	Formula	Parameters used in this work
Excluding	$lb \le {M_{ratio}} \le ub$	Lower bound lb = 0, upper bound ub = 1.6
Bounding	$\max (lb,\min (ub,{M_{ratio}}))$	Lower bound lb = 0, upper bound ub = 1.6
Logarithmic transformation	$\log \max (lb,{M_{ratio}})$	Lower bound lb = 0.1
Hierarchical estimation	see Fleming (2017)	

Determining these bounds based on the *M*_ratio_ distribution within an individual study will often be problematic, as such estimates are easily skewed by outliers in studies with typical sample sizes. I thus recommend using the bounds 0 and 1.6 proposed here, unless the sample size of a study allows for a sufficiently precise estimate of the distribution.

As a first regularization method, values of *M*_ratio_ were simply ‘bounded’ between the lower and upper bounds explained above. Second, a ‘logarithmic’ transformation of the *M*_ratio_ was tested, which was suggested by Fleming and Lau (2014) as a generic regularization method for ratio measures, giving equal weight to increases and decreases relative to an ideal metacognitive observer with log(*M*_ratio_) = 0. Note that taking the logarithm also requires the introduction of a positive lower bound, as the logarithm is undefined in the negative range. Here, 0.1 was chosen as a minimum. Third, a ‘hierarchical’ Bayesian estimation of *M*_ratio_ was evaluated (Fleming 2017), which effectively tames extreme values by means of a group prior.

### Measures of test**-**retest reliability

The test-retest reliability of metacognitive performance measures was evaluated both by means of the Pearson correlation and by means of a measure of absolute error, the normalized mean absolute error (NMAE). While the Pearson correlation is scale- and mean-invariant and thus quantifies to what degree the ‘pattern’ of results is similar across participants between two experimental sessions, the NMAE takes into account possible mean or scale shifts.

Let **x** and **y** be two vectors containing the metacognitive performance values of the *N* subjects for a test session (**x**) and a retest session (**y**). The Pearson reliability is computed as the standard sample correlation coefficient between paired data **x** and **y**. The NMAE of vectors **x** and **y** of length *N* is defined as follows:

(1) \begin{equation*}\textrm{NMAE}{\rm(x,y)} = {{{1 \over N}\sum\limits_{i = 1}^N {|{x_i} - } {y_i}|} \mathord{\left/ {\vphantom {{{1 \over N}\sum\limits_{i = 1}^N {|{x_i} - } {y_i}|} {\left( {{1 \over {2N}}\sum\limits_{i = 1}^N {|{x_i} - } \bar y| + {1 \over {2N}}\sum\limits_{i = 1}^N {|{y_i} - } \bar x|} \right)}}} \right. } {\left( {{1 \over {2N}}\sum\limits_{i = 1}^N {|{x_i} - } \bar y| + {1 \over {2N}}\sum\limits_{i = 1}^N {|{y_i} - } \bar x|} \right)}}\end{equation*}

While the numerator is the regular mean absolute error, the denominator is the mean absolute difference of each value and the average of the other session. The denominator ensures that the overall error is largely independent of the scaling of a measure, thus allowing comparisons between different metacognitive performance measures.

Note that the NMAE is not valid for the hierarchical *M*_ratio_ and was thus not computed for this metacognitive performance measure. The reason is that separate group priors are applied to the data of the test and retest set, which bias individual values to the respective group mean. The resulting distributions for test and retest have often considerable mean differences with individual values being tightly clustered around the respective means. While the overall mean difference is normalized for (denominator of the NMAE), the compression around the distribution means will lead to an artifactual reduction of the NMAE.

### Simulations

#### General model

I simulated artificial observers that were presented with stimuli pertaining to two stimulus categories. The task of observers was to identify the correct stimulus category, i.e. a binary choice task. I assume that percepts are subject to Gaussian sensory noise with standard deviation *σ*_s_:

(2) \begin{equation*}percept \sim N\left( { \pm {\mu \over 2},\ {\sigma _{\rm{s}}}} \right)\end{equation*}

where ±*μ*/2 are the stimulus means.

Using Bayes’ rule, the observer computes the choice probability *P* as follows:

(3) \begin{equation*}p = {1 \over {1 + {\textrm{exp}} \left( { - {{\mu \cdot percept} \over {\sigma _s^2}}} \right)}}\end{equation*}

The observer chooses stimulus category 2 whenever *P* ≥ 0.5 and else stimulus category 1. An auxiliary (choice-independent) confidence variable *c* ∈ [0; 1] is computed from this choice probability:

(4) \begin{equation*}c = \left\{ \begin{matrix} {2 \cdot (0.5 - p)} & {\textrm{if }}\ p \lt 0.5 \\ {2 \cdot (p - 0.5)}& \hspace{-20pt}{\textrm{else}} \\ \end{matrix} \right.\end{equation*}

However, the final confidence reports are subject to metacognitive noise described by a metacognitive noise distribution Μ with mode *c* and metacognitive noise *σ*_m_:

(5) \begin{equation*}confidence \sim M(c,{\sigma _m})\end{equation*}

The parameter *σ*_m_ of the metacognitive noise distribution Μ defines the spread of the distribution. For the simulations in this work, the Beta distribution was used, which is naturally bounded between 0 and 1:

(6) \begin{equation*}confidence \sim {1 \over {B(\alpha ,\beta )}}{x^{\alpha - 1}}{(1 - x)^{\beta - 1}}\end{equation*}

The used parameterization is *α *= *c* (1/*σ*_m_ − 2) + 1 and *β* = (1 − *c*)(1/ *σ*_m_ − 2) + 1, for which the Beta distribution has mode *c* and spread 0 <  *σ*_m_ ≤ 0.5 (note that *σ*_m_ is not a standard deviation). For the maximum value of *σ*_m_ = 0.5, the Beta distribution becomes the uniform distribution.

#### The relationship between type 1 performance and metacognitive performance

To assess the relationship between type 1 performance and metacognitive performance measures, type 1 and confidence data were simulated for different type 1 and 2 performance levels. One hundred different performance levels were evaluated by varying the sensory noise parameter *σ*_s_ from 0 (no sensory noise, i.e. perfect performance) and 5 (very high sensory noise, approximately chance-level performance). Six metacognitive noise levels were assessed by varying *σ*_m_ between 0 and 0.5 in steps of 0.1. For each pair (*σ*_s_, *σ*_m_), 1000 subjects with 10 000 trials each were simulated.

#### Test retest reliability

To evaluate the test-retest reliability of metacognitive performance measures, two experimental sessions were simulated for each subject. Type 1 performance and the number of trials in a session are expected to be critical factors for the test-retest reliability and were hence under special consideration. Specifically, the sensory noise parameter *σ*_s_ was varied to induce different levels of type 1 performance between 55% correct and 95% correct and the number of trials was varied between 25 and 1000 in steps of 25. For each pair (*σ*_s_, #trials), two independent experimental sessions of 100 subjects were generated. For each session, all metacognitive performance measures under consideration were computed. Reliability was assessed both by means of the Pearson correlation and the NMAE.

To estimate uncertainty, the entire procedure described above was repeated 300 times with different random seeds.

### Analyses of empirical data

#### Preprocessing

All studies were transformed to a homogenous format. Most importantly, as the computation of meta-*dʹ* requires discrete confidence ratings, a discretization procedure was defined. The maximum number of discrete confidence ratings was limited to 6, as this is the highest number that was used in a major proportion of studies. Trials were sorted into six bins on a percentile basis (aiming for a balanced number in all confidence bins) with the constraint that all bins must be unique. Negative confidence ratings, which some studies included to indicate that one is confident in being wrong, were floored at 1, the lowest confidence rating. Verbal confidence ratings were converted to appropriate numerical ratings (e.g. ‘difficult’ and ‘easy’ were converted to ratings 1 and 2) and fractional ratings (e.g. indicating the probability of being correct) were converted to integers. While it is clear that there is uncertainty in how to convert different confidence rating procedures to a common scale, this is an issue inherent to any particular scale already at the response stage, as there is generally little knowledge of how participants translate perceived levels of confidence to available rating options.

#### The relationship between type 1 performance and metacognitive performance

The relationship between *dʹ* and metacognitive performance measures in the Confidence Database was characterized in two ways. First, the overall linear slope between type 1 and type 2 performance across all subjects was computed by means of ordinary least square regression. This analysis shows whether there is a systematic linear trend between type 1 performance and each metacognitive performance measure. To derive a more fine-grained empirical function, averages for *dʹ* bins were computed, centred at values ranging from 0.3 to 2.9 in steps of 0.2 (window size: ±0.2) and standard errors within each bin were computed. The *dʹ* range was limited to a range of 0.1–3.1 (only a few subjects show even smaller or greater type 1 performance).

#### Test retest reliability

As it is possible that the test-retest reliability of metacognitive performance measures strongly depends on idiosyncratic characteristics of empirical data, I also evaluated the test-retest reliability of studies in the Confidence Database. As the number of trials per subject is a strong predictor of test-retest reliability, the studies of the Confidence Database were divided according to the split-half number of trials (5 bins: 0–200 trials, …, 800–1000 trials). For each subject, every second trial was assigned to the ‘test session’ and every other trial to the ‘retest session’. As in the simulation, I computed both the Pearson correlation coefficient and the NMAE (Equation 1) as measures of reliability.

## Supplementary Material

niab040_Supp

## Data Availability

The Confidence Database (Rahnev *et al.* 2020) is available at https://osf.io/s46pr. Code for all analyses and simulations is available under https://github.com/m-guggenmos/metameasure.
