# Supplementary material for: Measuring metacognitive performance: type 1 performance dependence and test-retest reliability
Source: Neurosci Conscious. 2021 Nov 25;2021(1):niab040. doi: 10.1093/nc/niab040 (PMC8633424; doi:10.1093/nc/niab040)
Supplement: niab040_Supp [file niab040_supp.zip › supplement_rev1.pdf]

Supplement to: Measuring metacognitive performance: type 1 performance dependence and test-retest reliability

Matthias Guggenmos[1]*

[1] Charité – Universitätsmedizin Berlin, corporate member of Freie Universität Berlin and Humboldt-Universität zu Berlin, Department of Psychiatry and Neurosciences, Charitéplatz 1, 10117 Berlin, Germany

* Corresponding author: Matthias Guggenmos (mg.corresponding@gmail.com)

Supplementary Figure S1. Simulation: test-retest reliability of additional metacognitive performance measures

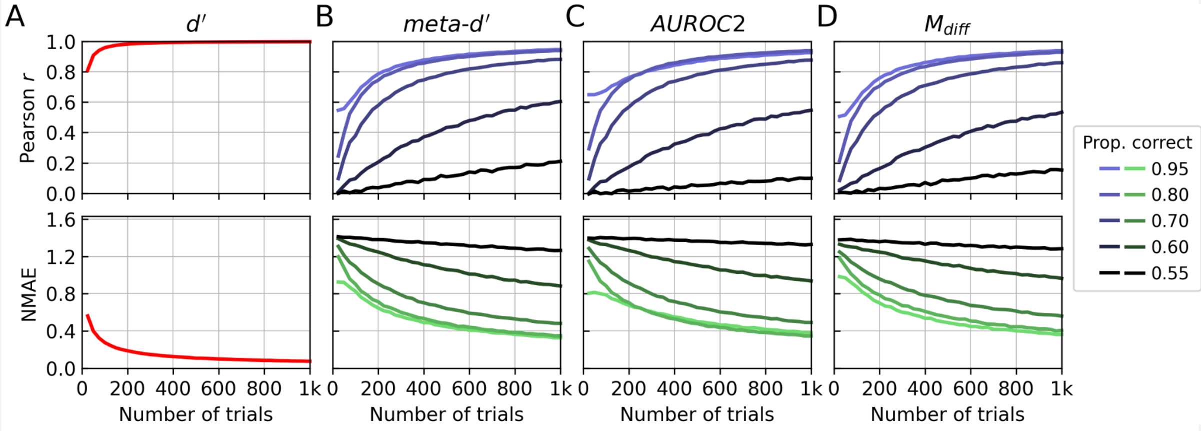

Supplementary Figure S1. Simulation: test-retest reliability of type 1 performance and additional metacognitive performance measures. Note that in contrast to metacognitive performance measures, the reliability of *d'* was computed across the entire range of simulated type 1 performance levels.

Supplementary Figure S2. Simulation: differences in test-retest reliability of regularized M_{ratio} variants

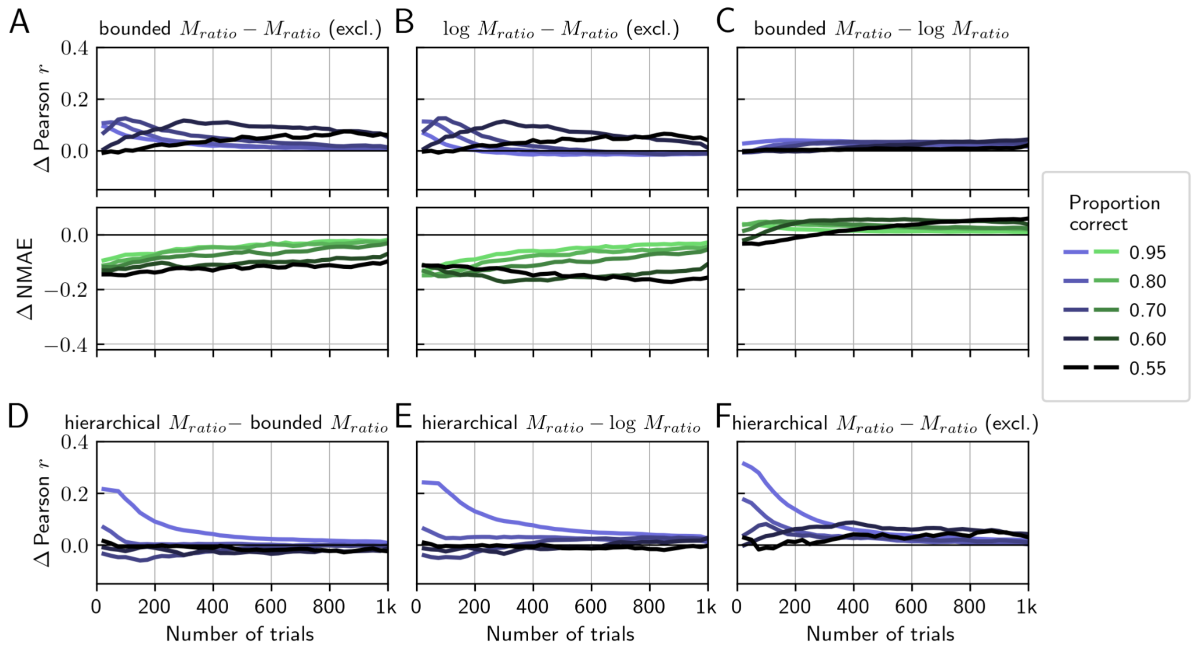

Supplementary Figure S2. Simulation: differences in test-retest reliability of M_{ratio} variants. Note that the NMAE is not valid for the hierarchical Mratio (see method section 3) and is thus not shown.

Supplementary Figure S3. Empirical test-retest reliability of type 1 performance and additional metacognitive performance measures

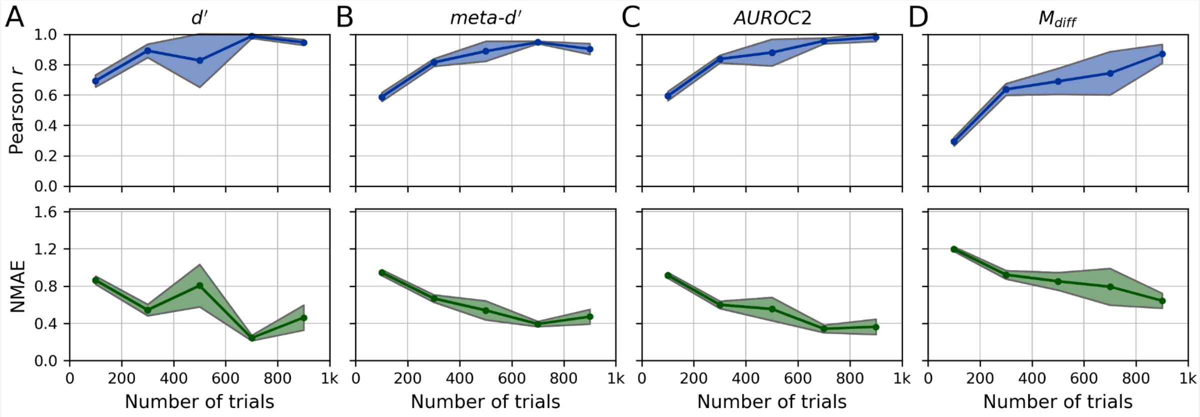

Supplementary Figure S3. Empirical test-retest reliability of type 1 performance and additional metacognitive performance measures. Test-retest reliability is computed either as the Pearson correlation coefficient or the normalized mean absolute error (NMAE) between test and retest trials. The x-axis denotes the average split-half number of trials for the Confidence Database studies (bins of 200 from 0-1000 trials), i.e. the number of trials per test and retest. In this way, the expected reliability for other/new studies with overall N trials can simply be obtained by looking up the reliability for x = N. Shaded areas indicate standard errors across studies.

Supplementary Figure S4. Type 1 performance dependency of M_{ratio} for other metacognitive noise distributions

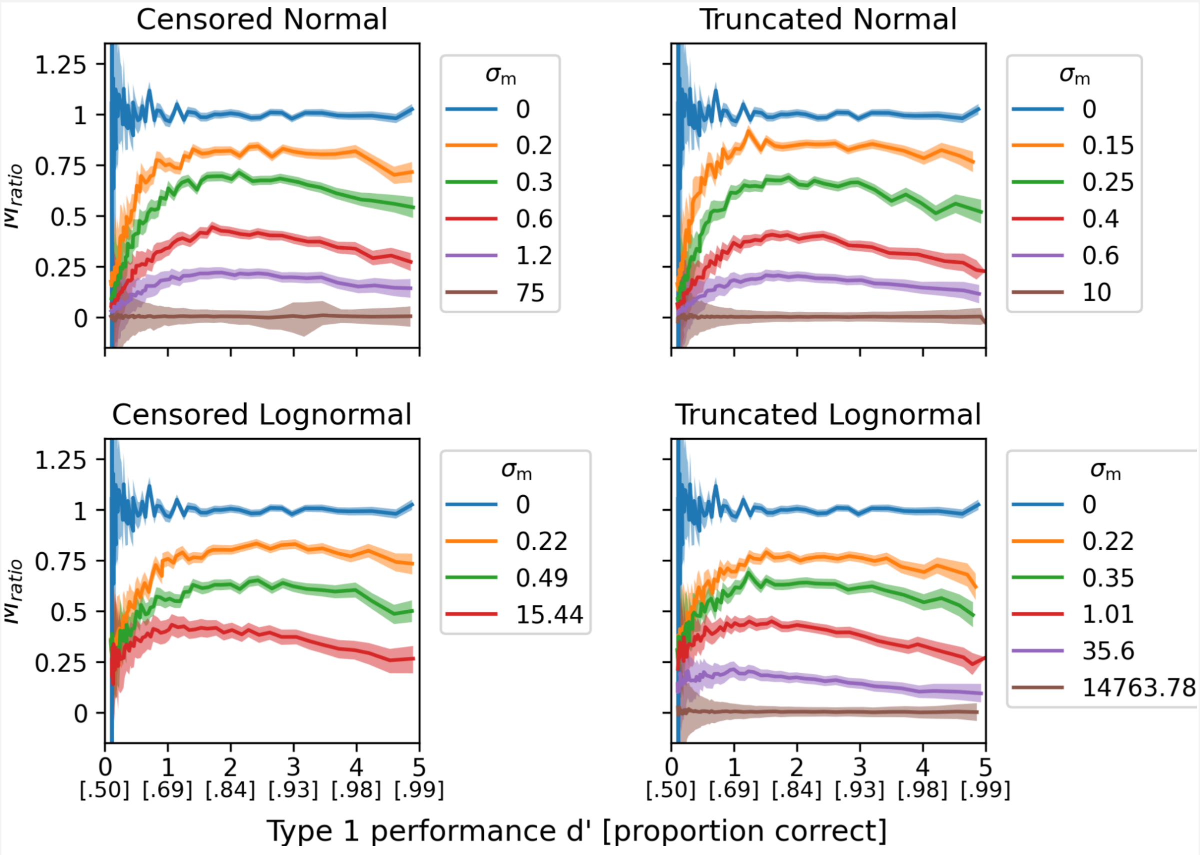

Supplementary Figure S4. Type 1 performance dependency of M_{ratio} for other metacognitive noise distributions. Normal and lognormal noise distributions were constricted to the range [0; 1] either via truncation or censoring. Truncating a distribution means to cut off the parts of the distribution outside the truncation points (here confidence < 0 and confidence > 1) and to renormalize the remainder of the distribution to 1. In the case of censoring, all values below 0 and above 1 are set to 0 and 1, respectively. The values for the parameter σ_m were chosen such that the average M_{ratio} values at each metacognitive noise level approximately match the average M_{ratio} values of the original Beta noise distribution. For the censored lognormal distribution, very high levels of metacognitive noise generate only maximal confidence ratings and are thus omitted. Shaded areas denote asymmetrical standard deviations.
